# Classification of Drivers' Workload Using Physiological Signals in Conditional Automation

**DOI:** 10.3389/fpsyg.2021.596038

**Published:** 2021-02-18

**Authors:** Quentin Meteier, Marine Capallera, Simon Ruffieux, Leonardo Angelini, Omar Abou Khaled, Elena Mugellini, Marino Widmer, Andreas Sonderegger

**Affiliations:** ^1^HumanTech Institute, University of Applied Sciences of Western Switzerland, Haute École Spécialisée de Suisse Occidentale, Fribourg, Switzerland; ^2^Department of Informatics, University of Fribourg, Fribourg, Switzerland; ^3^Bern University of Applied Sciences, Business School, Institute for New Work, Bern, Switzerland

**Keywords:** automated driving, classification, driver, workload, physiology, secondary task, machine learning

## Abstract

The use of automation in cars is increasing. In future vehicles, drivers will no longer be in charge of the main driving task and may be allowed to perform a secondary task. However, they might be requested to regain control of the car if a hazardous situation occurs (i.e., conditionally automated driving). Performing a secondary task might increase drivers' mental workload and consequently decrease the takeover performance if the workload level exceeds a certain threshold. Knowledge about the driver's mental state might hence be useful for increasing safety in conditionally automated vehicles. Measuring drivers' workload continuously is essential to support the driver and hence limit the number of accidents in takeover situations. This goal can be achieved using machine learning techniques to evaluate and classify the drivers' workload in real-time. To evaluate the usefulness of physiological data as an indicator for workload in conditionally automated driving, three physiological signals from 90 subjects were collected during 25 min of automated driving in a fixed-base simulator. Half of the participants performed a verbal cognitive task to induce mental workload while the other half only had to monitor the environment of the car. Three classifiers, sensor fusion and levels of data segmentation were compared. Results show that the best model was able to successfully classify the condition of the driver with an accuracy of 95%. In some cases, the model benefited from sensors' fusion. Increasing the segmentation level (e.g., size of the time window to compute physiological indicators) increased the performance of the model for windows smaller than 4 min, but decreased for windows larger than 4 min. In conclusion, the study showed that a high level of drivers' mental workload can be accurately detected while driving in conditional automation based on 4-min recordings of respiration and skin conductance.

## 1. Introduction

According to the National Highway Traffic Safety Administration (NHTSA), 2,935 fatal crashes occurred on U.S. roadways due to driver's distraction in 2017. This represents 9% of all fatal crashes (NHTSA, 2017). Performing a secondary task while driving is one cause that increases the risk to have an accident, among other factors such as fatigue, mood or demanding driving conditions. The latter lead to hazardous drivers states as named by Darzi et al. (2018). To solve that issue, car manufacturers aim at reducing the rate of accidents by proposing an increasing level of automation in cars to support the driver. According to the Society of Automotive Engineers (SAE) classification (SAE, 2018), the next generation of vehicles that will emerge on our roads will be conditionally automated cars, corresponding to Level 3 of the SAE taxonomy. At this automation level, the driver will no longer be in charge of the main driving task, neither monitoring the environment. However, the car alerts the driver that he or she has to take over control of the car when the automation is reaching its limit. The commonly accepted approach is sending a takeover request (TOR) to the driver (Kim et al., 2019). Various ways of alerting the driver are being tested (Petermeijer et al., 2017), such as visual, auditory and haptic alerts, or a combination of those. Thus, the driver must be ready to take over control at any moment during the ride. The role of the driver in such a situation will switch quickly from passenger behind the wheel to driver. Besides, on the basis of decisions taken by the authorities concerned, drivers could be allowed to engage in a Non-Driving-Related Task (NDRT) during periods of conditionally automated driving. The driver might be out-of-the-loop if he or she is engaged in a NDRT. It was recently defined by Merat as being “not in physical control of the vehicle, and not monitoring the driving situation, or in physical control of the vehicle but not monitoring the driving situation” (Merat et al., 2019). The engagement of drivers in a NDRT would distract them from the supervision of the environment for which they are responsible. They could be distracted visually, orally, cognitively, or bio-mechanically (Pettitt et al., 2005). These are not exclusive and drivers' could be distracted in different ways at the same time. The distraction induced by performing a NDRT using another sensory channel might also increase the mental workload (MWL; Mehler et al., 2009).

Previous studies showed that performing NDRTs that involve various modalities affect the gaze behavior and takeover performance of drivers (Nakajima and Tanaka, 2017; Wandtner et al., 2018). To address this issue, Parasuraman et al. (2000) suggested that “well-designed information automation can change human operator mental workload to a level that is appropriate for the system tasks to be performed.” If we want the drivers to safely engage in NDRTs, it is crucial to find a way to measure continuously their state and use this information to dynamically support the driver. Various types of measures that depict the operator's state could be used such as performance, subjective or physiological measures. Under real situations, it might not be the best option to rely on subject ratings for adapting the level of automation. Driving data were suggested in previous studies to show drivers' distraction and elevations of MWL induced by a NDRT in manual driving (Engström et al., 2005). However, this source of data cannot be used in conditionally automated driving since the car is performing the main driving task most of the time, except during takeover situations. Previous research has shown that increases of MWL and cognitive distraction can easily be detected with cameras using eye-tracking, face-based or EEG features (Li and Busso, 2013; Hogervorst et al., 2014). For practical issues, a EEG headset would not be comfortable to wear for drivers, therefore, we are not considering this signal in this study. When the driver's gaze is toward the windshield and facing the camera, these features could be useful for predicting driver's workload continuously. However, the driver's gaze often changes direction in the car. Thus, it may be difficult to use only these data sources in real driving conditions to measure driver cognitive load.

In this context, physiological signals seem to be the best option to evaluate continuously and non-intrusively changes in MWL of drivers while performing a secondary task in conditionally automated driving. Recent advances in technology allow for a recording of physiological signals with embedded sensors that drivers could wear in real-world environments such as wristbands, smartwatches, or smart clothes (Sonderegger, 2013; Angelini et al., 2014). Features computed from raw physiological signals could be used to classify the driver's state using recent machine learning techniques. This information could be used to adapt either the automation level or the interaction level between the driver and the car. Such a system would help reducing fatalities due to bad takeover behavior and performance and therefore increase safety on roads. Besides, this system could also increase the user experience in automated cars because drivers would be able to engage in their favorite activity during the ride. This is because, based on such information, an automated system could adapt the type of warning (e.g., display of a loud and startling sound in case of low activation of the driver vs. smooth visual hint in case of situations of high activation) or even decide not to send out a TOR because it calculated that there is not enough time to safely take over control under given conditions (e.g., travel speed, distance to the object, state of the driver).

## 2. Related Work

### 2.1. Mental Workload

Being one of the most widely invoked concepts in human factors and ergonomics, MWL represents a topic of increasing importance in research and practice (Young et al., 2015). MWL can be explained in terms of the balance between the demands of a situation (task and environmental context) and the resources an individual has available to overcome the situation (Wickens, 2008). While task demands are generally referred to as stress, strain describes the impact of task demands on the human (Schlegel, 1993). MWL is generally defined as a multidimensional construct which is determined by task characteristics, operator characteristics (e.g., attentional resources, skills), and the environmental context (Young et al., 2015). One of the main reasons for the increasing interest in MWL lies within its link to human performance and hence the possibility to identify suboptimal workload conditions that might lead to stress, errors and incidents in the driving context (Brookhuis and De Waard, 2001). It is generally agreed upon that MWL can be considered a basic precursor of stress, errors, and accidents—it has been difficult however to establish an exact relationship between the concepts, which mainly might be due to difficulties in the accurate measurement of MWL (Young et al., 2015).

Three main approaches have been put forward for assessing MWL, including measures of task performance (primary and/or secondary task), subjective ratings based on questionnaires and physiological measures (Gawron, 2019). The first approach is based on techniques measuring task performance on a primary and a secondary task. While generally an acceptable level of performance in the primary task can be maintained in high workload conditions, performance on the secondary task is highly correlated with MWL since the secondary task is associated with the spare capacity unused for completion of the primary task (Young et al., 2015). The primary-secondary task paradigm has been shown to be a good indicator of MWL in experimental research. Its implementation however is linked with some rather severe drawbacks (e.g., artificial setup of test environment with a high need for standardization and control of the task scenarios; Fisk et al., 1986). In some driving studies, the performance on the primary task was measured using driving data (longitudinal and lateral parameters) and the secondary task performance was used as an indicator of MWL (Engström et al., 2005; Mehler et al., 2009). The second approach is to assess MWL as subjective state based on subjective ratings. This implies the assumption that humans are capable of evaluating and expressing the level of MWL they experience in a specific task after task completion. Some widely used questionnaires for measuring MWL subjectively are the NASA-TLX (Hart and Staveland, 1988) or the Rating Scale Mental Effort (Zijlstra and Doorn, 1985). Task load questionnaires are easy to apply and interpret but come along with some methodological issues which are due to the subjectivity of the measure and the retrospective bias of post-task assessments (Bulmer et al., 2004). The third approach to measure MWL is the assessment of physiological indicators. In this regard, two groups of physiological indicators can be differentiated, indicators of the autonomic nervous system and indicators of the central nervous system. Cardiovascular indicators (e.g., heart rate and heart rate variability) as well as electrodermal activity (e.g., tonic and phasic skin conductivity) are often referred to as useful indicators of MWL in research (De Waard, 1997). However, considerable drawbacks for the assessment of MWL via physiological parameters are the troublesome procedure of applying electrodes, the generally rather high signal to noise ratio as well as the interfering influence of physical activity (Huigen et al., 2002). As mentioned before, it is difficult to measure MWL using task performance or subjective ratings in real-world conditions. In this study, these measures are used to control the success of MWL manipulation. Besides, the use of physiological signals as a potential source of data for measuring MWL in conditionally automated driving is explored. In the present study, we concentrate on measurements of the autonomic nervous system for classifying drivers' workload. This is because we consider EEG or near-infrared spectroscopy are being less suitable under real-world conditions since drivers might be averse to wearing a headset. Besides, drivers' gaze can constantly switch between the windshield, the dashboard and potentially a tablet or a smartphone held in the hands during conditionally automated driving, which makes it challenging to continuously capture this feature. However, we are convinced that these measures could also represent interesting indicators and should be considered in future research.

### 2.2. Definition of Physiological Indicators

#### 2.2.1. Electrodermal Activity (EDA)

The first selected physiological signal is the EDA, which is defined as the changes in the electrical conductivity of the skin, caused by the fluctuations of sweat in glands regulated by the autonomic nervous system (Cacioppo et al., 2007). The latter can be declined in two main components. One feature which can be derived from EDA data is the tonic level of EDA which refers to the slow-acting components of electrical activity such as the mean level of EDA or slow climbing and decreases over time. The most common measure of this component is the skin conductance level. Changes in this measure reflect general changes in arousal (Cacioppo et al., 2007). The second component is the phasic component of EDA, which refers to fast-changing properties of the signal. It is measured with the Skin Conductance Responses (SCRs). Previous research suggested that both components are important and may rely on different neural mechanisms (Cacioppo et al., 2007). Phasic SCRs can be distinguished into two categories called non-specific SCRs (NS-SCRs) and event-related SCRs (ER-SCRs). The first one gathers responses occurring in the absence of identifiable eliciting stimuli, while the second one characterizes subjects' electrodermal reaction to stimuli. One commonly used indicator is the frequency of NS-SCRs, which is generally between one and five per minute at rest, and more than 20 per minute in periods of high arousal. To characterize ER-SCRs, indicators such as latency, amplitude, rise time and half recovery time are usually used (Boucsein, 2012). The same indicators can be calculated identically for NS-SCRs, except for latency which requires a time-stamped triggered event to be calculated.

#### 2.2.2. Electrocardiogram (ECG)

The second selected physiological signal is the ECG. Various indicators can be computed based on ECG data such as the heart period (the time interval between successive heart cycles) and the heart rate variability (HRV; Camm et al., 1996). The heart period is also known as the inter-beat interval (IBI). Another widely used metric to evaluate the cardiac activity is the heart rate (HR) which corresponds to the number of heartbeats per unit of time, usually per minute. HRV is a general term that refers to time changes in IBI. These measures are used as indices of autonomic nervous system regulatory activities and have been related to individual differences in attention and cognition in various groups of populations (Cacioppo et al., 2007). Previous studies showed that mental effort is related to changes in cardiovascular state (Aasman et al., 1987; Bernston et al., 1993) and more specifically in HRV (Mulder, 1992). The HRV can be quantified by two different categories of methods. The first method is the time-domain method. This category contains both statistical and geometric measures, depicting the variability of time between heartbeats (Camm et al., 1996). Malik and Terrace (1996) recommend indicators to use in this regard that are the standard deviation of IBI (SDNN, for estimating the overall HRV), the HRV triangular index (an estimate of the overall HRV), the standard deviation of IBI calculated over short periods (SDANN, an estimate of long-term changes of HRV) and the square root of the mean squared differences of successive IBI (RMSSD, an estimate of short-term components of HRV). While some specialists in the field advise calculating common statistical time-domain HRV measures such as SDNN or RMSSD using at least a 5-min ECG recording (Camm et al., 1996; Malik and Terrace, 1996), other investigations have utilized ultra-short term measures (below 5 min) (Shaffer and Ginsberg, 2017). Studies showed that 10 s for HR, 30 s for RMSSD and 60 s for other metrics such as pNN50 could be enough to get a reliable measure of cardiac activity (Salahuddin et al., 2007; Baek et al., 2015). Other metrics such as SDANN and the HRV triangular index require long-time monitoring (at least 20 min, preferably 24 h; Malik and Terrace, 1996). The second method to evaluate changes in HRV is the frequency-domain method. Power spectral density method provides information on how power (e.g., variance) distributes as a function of frequency. Three main components can be distinguished in periods of two to 5 min of recording, including the Very Low Frequency (VLF; below 0.04 Hz), the Low Frequency (LF; between 0.04 and 0.15 Hz) and the High Frequency (HF; between 0.15 and 0.4 Hz; Malik and Terrace, 1996). More recent researches suggest that 20–90 s could be enough to evaluate the components of HF and LF (Salahuddin et al., 2007; Baek et al., 2015). However, the use of VLF should be avoided to interpret recordings shorter than 5 min. The ratio LF/HF is also an indicator used to emphasize the behavior of the two main branches of the autonomic nervous system.

#### 2.2.3. Respiration

The third physiological signal recorded in this study is the respiration of drivers. The respiratory system is complex and sensitive to other psychological variables (Cacioppo et al., 2007). Respiration forces the chest to expand and this movement of chest expansion can be measured by piezoelectric sensors. The respiratory system is linked with other muscles of the body as well as with the nervous system. Under ideal conditions, the respiratory activity is regular and harmonious but it can be perturbed when experiencing stressful situations. Previous research showed that respiration influences both EDA and heart activity (Cacioppo et al., 2007). Several measures can be extracted based on the information provided by breathing transducers such as the breathing rate (BR), which corresponds to the number of breathing cycles per minute. Inspiratory and expiratory volumes and durations, the ratio of both, and the complexity of the signal (through spectral analysis) are also measurements that can be derived from the raw breathing signal.

#### 2.2.4. Respiratory Sinus Arrhythmia (RSA)

Heart rate changes as a function of the respiratory cycle. This phenomenon is called respiratory sinus arrhythmia. RSA has become of great interest in recent years since the tight coupling of both signals can be used as an index of the vagal control of the heart (Cacioppo et al., 2007). Many factors influence RSA such as posture, age, or activity. The main measure is the magnitude of RSA but both frequency and time domain methods can be used as well since they showed similar results.

### 2.3. Influence of MWL on Physiological Measures

Previous studies already investigated the influence of increased MWL induced by cognitive tasks on the physiological state of subjects. The goal is to summarize previous findings in order to get a better appreciation of the expected results in this study. Studies that manipulated MWL by administering a secondary NDRT to drivers were reviewed, as well as studies that manipulated MWL with a cognitive task that subjects had to perform on a computer under experimental laboratory conditions. Previous research showed that the EDA level increases with increasing task difficulty. It has been shown for subjects performing oral or auditory tasks while driving in the real field (Collet et al., 2009) or in a simulated environment (Mehler et al., 2009, 2012). The same effect was found for a visual task performed while driving in both real field and simulated environments (Engström et al., 2005) or on a computer (Ikehara and Crosby, 2005). However, no effect of task difficulty was found on EDA for an auditory task performed in a driving simulator (Engström et al., 2005). A possible explanation was provided by Mehler et al. (2012) in their follow-on study for this non-consistent effect of incremental difficulty of task on EDA in the study of Engström et al. (2005). Some participants might have disengaged from the task when performed at high levels of difficulty, resulting in a lower physiological activation. This shows the high importance of controlling the performance of the participants with regard to the secondary task. For measures describing the cardiac activity, HR and IBI were also shown to be sensitive to increased task difficulty. IBI decreases (e.g., HR increases) with increasing difficulty of the visual task performed in both simulated and real environment (Engström et al., 2005). The same effect was found for auditory tasks (with verbal prompt or not) performed under real driving conditions (Engström et al., 2005; Mehler et al., 2009). Collet et al. (2009) found similar results since HR of participants increased when performing various oral and auditory tasks while driving. An effect of increased task demand induced by the environment in simulated driving was also found on HR and frequency-based HRV measures (Brookhuis et al., 2004; Brookhuis and de Waard, 2010). In addition, the respiratory activity of subjects is also sensitive to the performance of the auditory prompt-verbal response “n-back” task while driving (Mehler et al., 2009). This study showed a plateau effect between the 1-back and 2-back conditions for BR and EDA, suggesting that it might be difficult to distinguish two different levels of high cognitive workload using physiological measures. Subsequently, Mehler et al. (2012) found that the seeming plateau effect in the earlier study was an artifact of the methodology employed and that when the order of task difficulty is randomized, significant differences in EDA level between the 1-back and 2-back were observed, confirming that the mean EDA level increases with task demand. In summary, previous studies led in various experimental settings already showed that changes in driver's workload can be measured using physiological signals. Some significant results were found in both real field and simulated environments (Engström et al., 2005), although the simulation was probably less realistic than it is now. Some indicators such as the mean EDA level or IBI can be used to measure changes in workload. Increasing task difficulty lead to increasing MWL, which goes with reduced IBI (e.g., higher HR), increased mean EDA level and increased BR. Some of these measures showed to vary when engaging in tasks involving different sensory channels such as auditory, oral or visual tasks. The fact that a speech-based cognitive task increases EDA, HR, and BR (Collet et al., 2009; Mehler et al., 2009) is particularly relevant for our study. We expect that these indicators will play a significant role in the classification of drivers' condition.

### 2.4. Classification of Workload

Our contribution is to classify drivers' workload using physiological measures during conditionally automated driving. In this regard, the classification results and procedure from previous studies, including the type of chosen physiological signals, the features generation and selection techniques, the selected classifiers, the validation techniques, or the number of classes to predict are reported. In this section, we reviewed studies for which the experimental task was the accomplishment of a NDRT during manual driving periods (real or simulated driving) or the performance of a single cognitive task in a laboratory. For each study, the method employed by authors to perform the classification is described.

Ferreira et al. (2014) asked two groups of adults (young vs. old) to perform two different cognitive tasks on a computer, testing their perceptual speed and visio-spatial cognitive processing capabilities. Each group performed three blocks of these two tasks, with two different difficulty levels of the tasks. One hundred and twenty-eight features were extracted from the EEG, ECG, EDA, heat flux, and respiration raw signals. Features were computed from 10 and 60-s segments using sliding windows with a step of one second. An inter-subject classification achieved results from 64 to 86% accuracy to distinguish two difficulty levels, depending on the task, the age group and the time window used for classification. The best scores were achieved mostly with data collected from young participants but with a high variation.

Haapalainen et al. (2010) administered six elementary tasks to 20 young subjects on a computer. Tasks were asking for visual perception and cognitive speed. A Naive Bayes classifier had to choose between two levels of cognitive load (low vs. high) using features derived from non-overlapping segments of psychophysiological measures during the tasks. Features such as statistical indicators of pupil diameter, GSR, heat flux, mean absolute deviation of ECG, EEG power values, two mental state outputs, heart rate and time-based HRV features were calculated. A leave-one-out approach was used for validation. Finally, the authors averaged the classification results across all participants, using the best feature from each sensor. An average of 76 and 71.4% of accuracy was achieved with respectively the heat flux and mean absolute deviation from ECG. An accuracy of 81.1% was achieved by combining both features. Besides, the classification with EDA as an input feature showed the lowest performance.

In another study led by Hogervorst et al. (2014), 14 participants had to perform the visual n-back task on a computer at different levels of difficulty (rest, 0, 1, and 2-back). Each participant did 8 epochs of 2 min of that task in the 3 difficulty levels. Features such as frequency-based indicators from EEG, mean EDA level, time-based HRV indicators, breathing frequency, and eye-related indicators were used. The best classification accuracy reached was a little over 90% for distinguishing high (2-back) and low (0-back) workload on the basis of 2 min segments with all indicators. The breathing frequency was the most useful physiological measure for classifying workload level. Using only physiological features, the best accuracy achieved was around 75% to distinguish 2-min segments of 0-back and 2-back task using the support vector machine classifier. This score decreased slightly under 70% when using 30-s segments for the classification.

Son et al. (2013) collected driving, physiological and eye movement data of 30 participants performing the auditory n-back task while driving. Task difficulty was varied on three levels (0, 1, and 2-back task) for a duration of 2 min each. HR and skin conductance level were used as physiological features. Ten-second windows across all 2-min windows were used to compute the features. A support vector machine classifier with a nested cross-validation technique was used to classify periods of normal driving and dual-task periods (NDRT and driving). The heart rate showed the best accuracy as a single feature to classify workload with 80% accuracy. In addition, all models with the two physiological inputs (HR and skin conductance level) obtained at least 82.6% accuracy.

A recent study led by Darzi et al. (2018) aimed at identifying the causes of hazardous driver states, using a combination of driver characteristics, vehicle kinematics, and physiological measures. 21 drivers were asked to perform four 45-min sessions of simulated driving. Each driving session contained eight scenarios with changing weather, traffic density and NDRT. The classification of cell phone usage periods only with physiological data is the most relevant result for our study because it is close to what we are trying to achieve in this experiment, except that it is in the context of conditionally automated driving. During cell phone use, participants indicated to have a higher MWL with regard to NASA-TLX (NASA Task Load Index, Hart and Staveland, 1988) results. Seventeen features were computed from four physiological signals (ECG, EDA, respiration, and temperature) from each 4-min scenario. It included time-based and frequency-based HRV measures, skin temperature, indicators of tonic and phasic EDA and respiration rate and variability. To automatically classify drivers' condition, the support vector machines, logistic regression and decision trees were selected as classifiers. For the physiological features, the baseline value was subtracted to driving value and then normalized using the minimum and maximum during a session. Only significant features to the stepwise forward feature selection (threshold of 0.05) were selected and the leave-one-out validation method was employed. Only with physiological features, classifiers were able to detect that participants used the cell phone while driving with a 81.8% accuracy. The most useful physiological features for classification were the mean breathing rate and the absolute value of the gradient of the ECG signal.

Another main study in this domain had been led by Solovey et al. (2014). They conducted two field studies with 20 and 99 drivers to classify workload of drivers only with physiological data using respectively a subject-dependant and a subject-independent classification approach. Once again, the n-back task was used to manipulate drivers' workload (auditory prompt and verbal answer with digits). Various size of time windows (10–30 s) and overlapping factors (0–75%) were tested. Statistical measures of HR, skin conducatance level and vehicle velocity were used for classification. For the subject-dependant classification, accuracies around 75% were achieved with all classifiers (except for the k-Nearest Neighbor one) using all features. The model accuracy did not decrease much using only HR features for classification. For the subject-independent classification, accuracies around 90% were achieved only with physiological measures. The additional driving features did not increase accuracy, suggesting that physiological measures alone have a great potential for classifying drivers' workload in automated driving (where driving features are not available). Logistic Regression, Multilayer Perceptron and Naïve Bayes classifiers were the most efficient ones. Increasing the time widows for computing features increased the accuracy, with the best accuracy achieved with a 30-s time window. However, the overlapping factor did not affect the accuracy of the system.

Finally, Le et al. (2018) recently considered using near-infrared spectroscopy to similarly classify drivers workload being cognitively distracted by a NDRT. Again, the n-back task was chosen for manipulating workload and 6 features were computed from the sensor data. Five-fold cross-validation and a principal-component analysis were applied to data before the final classification. Five different classifiers were tested to classify three workload levels (driving only vs. driving + 1-back vs. driving + 2-back), including decision-tree, discriminant analysis model, logistic regression, support vector machine and nearest neighbor classifiers. Scores above 88% accuracy were achieved for subject-dependant classification and between 84 and 90% for subject-independent classification. The results obtained in this study are very promising the results are obtained for classifying three levels of workload compared to other studies that classified only two levels. However, the sensor was placed on the forehead of drivers, who may not be willing to wear such a device under real driving conditions.

To summarize the findings from previous studies, the main results of each article are presented in Table 1. Overall, decreasing the time window for computing physiological measures showed to decrease accuracy. Apart from psychological features such as EEG, some of the best physiological features to classify MWL were the breathing rate, HR or the mean absolute deviation of IBIs. Another main result to consider is that the models developed in several studies always benefited from sensor fusion. This leads to a compromise to classify the driver's condition. Previous research reviewed here raises a fundamental issue if we want to implement such systems in vehicles. The stake is to find the best trade-off between the number of physiological signals, features and time window to build a reliable and robust model (e.g., high accuracy with low variance). If too many signals are selected, it is difficult for the driver to wear many sensors under real driving conditions. Also, if a time window of a few minutes is needed to get an acceptable accuracy, this takes us away from real-time MWL assessment and therefore makes an implementation of such a system less credible.

**Table 1:** Summary of state of the art.

**References**	**Physiological features**			**Best classifier**	**Best performance (accuracy)**	**Best features**
	**ECG**	**EDA**	**RESP**			
Ferreira et al. (2014)	Yes	Yes	Yes	QDA	86% (60 s)	EEG, respiration rate
Haapalainen et al. (2010)	Yes	Yes	No	NB	83.70%	Heat flux, HRV
Hogervorst et al. (2014)	Yes	Yes	Yes	SVM	75% (2-back vs. 0 back, 120 s)	EEG, respiration, RMSSD
Son et al. (2013)	Yes	Yes	No	SVM	82.9%	HR, skin conductance
Darzi et al. (2018)	Yes	Yes	Yes	LR	82.3% (cell phone or not)	ECG gradient, respiration rate
Solovey et al. (2014)	Yes	Yes	No	MLP	75.7%; HR only : 74%	
	Yes	Yes	No	LR	90% (30 s); HR : 80–85%	
Le et al. (2018)	No	No	No	DT	89.91%	

*QDA, Quadratic Discriminant Analysis; NB, Naïve Bayes; SVM, Support Vector Machine; LR, Logistic Regression; MLP, Multilayer Perceptron; DT, Decision Tree*.

## 3. Current Study

In this paper, we propose a solution that classifies the driver's MWL (high vs. low) based on physiological data during conditionally automated driving. In particular, the following contributions are made:

- Creation of a dataset containing three physiological signals (ECG, EDA, and respiration) of 90 subjects in the specific context of conditionally automated driving in a simulator.
- Manipulation of drivers' MWL through a verbal cognitive task with a rigorous experimental approach. The selected task is similar to a task that drivers might engage in under real driving conditions (e.g., talking on the phone or to another passenger).
- Validation of the success of workload manipulation by means of the widely used questionnaire NASA Task Load Index (NASA-TLX, Hart and Staveland, 1988).
- Training of three different classifiers to predict drivers' condition, using a k-fold cross-validation approach.
- Evaluation of the effect of selected physiological signals and segmentation level (e.g., size of time windows used to compute features) on performance of classifiers.

Our approach differs from previous studies because it investigates the change of MWL of drivers in the specific context of conditionally automated driving. With the future rise of automated driving, it is important to validate that the results of previous findings are consistent with the increase of drivers' MWL at higher levels of automated driving. Besides, the measures used to classify drivers' MWL differ from some previous studies that used eye-tracking, EEG or driving features. In this study, only physiological signals that can be collected in real-world conditions using smart embedded sensors are used. Therefore, findings from this study are relevant to the potential use of physiological signals for detecting changes in MWL of drivers in future conditionally automated cars.

## 4. Materials and Methods

### 4.1. Experimental Method

#### 4.1.1. Participants and Experimental Design

90 young participants (24.15 ± 5.95 years old) within a tight age range were recruited for this study. 40 of them identified themselves as male, 49 as female and 1 as other. Participants were mainly students. All participants were required to hold a driving license and be of good general health. Students received course credit for their participation. All the research and measurements followed the tenets of the Helsinki agreement and written informed consent was obtained from all participants.

**Figure 1:**
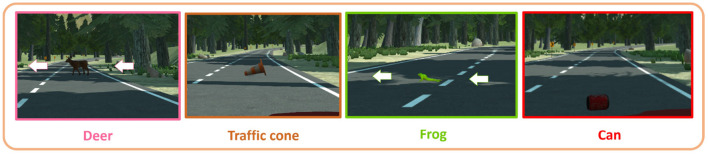
Takeover situations.

The experimental design was a 2 × 6 mixed-design with the task difficulty as a between-subject variable (secondary task vs. no secondary task) and the takeover situation as a within-subject variable (deer vs. traffic cone vs. frog vs. can vs. false alarm 1 and 2). The cognitive NDRT that half of the participants had to perform was a verbal cognitive task named oral backward counting (Siegenthaler et al., 2014; Krueger et al., 2019). It consisted of counting backwards for 20 min from 3,645 by step of 2. This artificial task was chosen because it is a continuous task similar to a discussion on the phone or between passengers in the car. With such task, a higher level of MWL was continuously induced over a long period of time. This gave the possibility to investigate the effect of segmentation on physiological signals. Also, the engagement in such difficult task could be measured. The six takeover situations included four obstacles that led to taking over control: a deer and a frog crossing the road, as well as a traffic cone and a can standing on the track (Figure 1). Participants also received two false alarms. They could choose to take over control if they estimated that the situation was dangerous for them and the car. The takeover situations were implemented in the scenario to make it more realistic and engaging for participants. However, the effect of the takeover situation on the physiological state of subjects is not presented in this work.

#### 4.1.2. Material and Instruments

**Figure 2:**
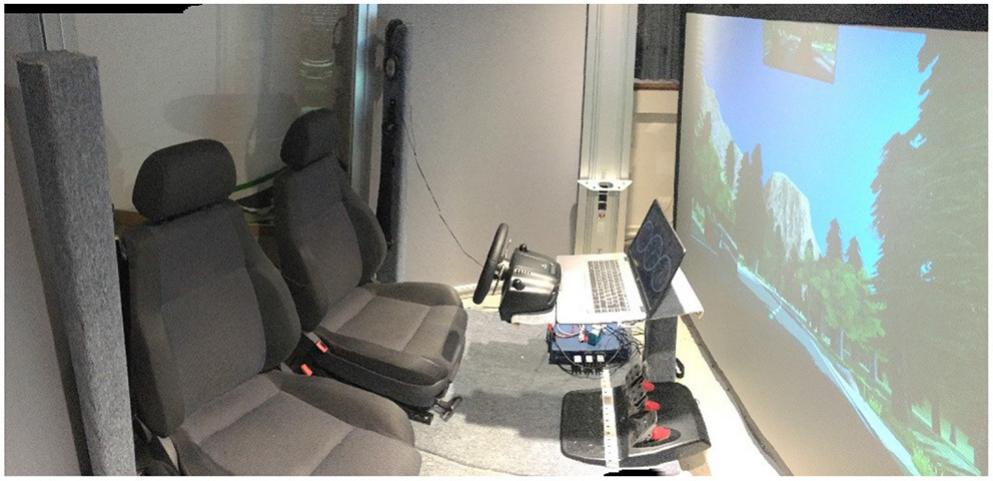
The driving simulator.

The experiment was conducted on a fixed-base simulator, as shown in Figure 2. It is composed of two adjacent car seats with seat belts and a Logitech G27 steering wheel with the gas, brake and clutch pedals. The clutch was not used in this study since the car used in the simulation had an automatic gearbox. The orientation and the longitudinal position of the seats toward the steering wheel were adaptable like in a real car. All this structure was installed in front of a large screen where the driving simulation software was back-projected with a projector (model Epsilon EH-TW3200). Two speakers were set up behind the seats to immerse the driver in the simulated driving environment. A cabin-like room with low ambient lighting contained all of this installation. The driving simulation used GENIVI software, developed with Unity by a consortium of car manufacturers. The scenario used for the experiment was a replication of the Yosemite National Park (USA) and included conditionally automated driving features. It was modified to add the obstacles triggered by the experimenter, leading to six takeover requests throughout the driving session. During the experiment, the Biopac MP36 hardware recorded the physiological signals of drivers, at a sample rate of 1,000 Hz. Appropriate parameters were used for each channel. A digital low pass filter with a frequency of 66.5 Hz and a Q factor of 0.5 reduced the noise of the three signals. For the EDA and RESP signals, the filter had a respective gain of 2,000 and 1,000. The SS57LA and SS2LB lead sets (Biopac) with disposable Ag/AgCl pre-gelled electrodes (EL507 and EL503, Biopac) respectively collected the EDA and ECG of participants. The SS5LB respiratory effort transducer (Biopac) recorded the respiration via chest expansion and contraction. The voice of participants assigned to the manipulation group was recorded by an audio recorder placed behind the dashboard.

#### 4.1.3. Measures

Physiological signals of participants were recorded throughout the whole experiment, including ECG, EDA, and RESP. Based on these data, physiological indicators were calculated. The creation of features from these indicators is presented later in the article (section 4.2.1). The subjective workload was assessed using the widely used questionnaire NASA-TLX (Hart and Staveland, 1988). It is a 6-item questionnaire where participants report their subjective level of workload during a task. After their experience in the driving simulator, participants were asked to rate their workload during the main driving session. The scale was modified due to visualization problems on the questionnaire. Hence, each item rated on a 11-point scale, from 0 to 10 (0 = Low, 10 = High). The mean score of the six items was computed to create a global score of MWL rating from participants. To ensure that participants were engaged enough in the NDRT throughout the driving session, we also measured NDRT performance of the participants. The frequency of orally spoken number (i.e., the number of orally spoken numbers per minute) was calculated from recordings obtained with the voice recorder. From times to times, participants stopped counting since the task was monotonous. For that reason, we also counted the number of times the experimenter asked the participant to resume counting.

#### 4.1.4. Procedure

After initial instructions about the experiment, participants answered a questionnaire containing socio-demographic questions (i.e., age, gender, driving experience, accidents, etc.). To record the physiological signals, the experimenter attached electrodes and the respiration belt to the participants' body. Three electrodes were attached to record the ECG, two above both ankles and one at the right wrist. For the EDA, two electrodes were attached to the index and middle finger of the right hand of participants. Then, the experimenter asked them to take a seat in the simulator. The experiment took place in three distinct periods. Oral instructions were given by the experimenter before each period to ensure that participants understood the experimental procedure. As described in the section 4, the three periods took place in the same scenic environment. During the first period, participants had to monitor the environment of the car while it was driving in conditional automation for 5 min. They were told that no takeover could be requested during this period. Indicators computed during this phase corresponded to the physiological baseline of participants.

**Figure 3:**
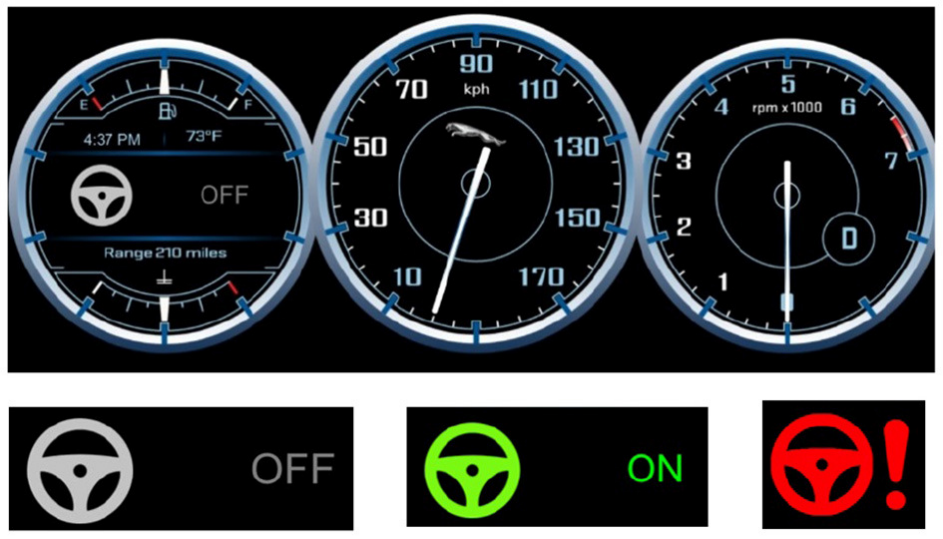
Upper part: The dashboard displaying the icon indicating the autopilot mode, the speed of the car and the number of engine's revolutions per minute. Bottom-left: gray icon—Autopilot OFF, Bottom-middle: green icon—Autopilot ON, Bottom-right: Red icon—Takeover Request (TOR).

The second period served as a practice session for the participants. During 5 min, they could familiarize with the takeover process as well as with the driving functions of the simulator (e.g., sensitivity of the steering wheel, gas and brake pedal etc.). Before starting, the experimenter reminded the subjects that they were driving a level 3 vehicle. The meaning of icons showing the state of the autopilot on the dashboard was explained to the driver (cf. Figure 3). For each TOR, the simulation displayed a red icon on the dashboard and played an audio chime in the speakers. The experimenter also explained how the participants could take over control of the car, either by steering the wheel, braking or pressing the upper-right button placed on steering wheel. For the practice session, drivers were told that three false alarms would be triggered to become familiar with the process. After the three false alarms were triggered, the experimenter made sure that participants understood the process. Then, they had the chance to drive manually until the end of the 5 min. This study does not include the analysis of data during the practice drive.

The third period consisted of the main driving session that lasted 20 min. The experimenter reminded the participants to take over control of the car only in a situation that they considered dangerous for themselves and the vehicle. They had to react accordingly to six TORs. Each one was randomly triggered between 1 min and a half and 4 min after the previous TOR. The randomization of time between takeover was implemented to avoid an expectation effect. Once participants gained control over the critical situation and considered it as safe again, they were instructed to reengage the autopilot. To do that, they had to position the car in the center of the right lane and press a button on the steering wheel. In addition, half of the participants had to perform the speech-based cognitive secondary task while the car was driving. At the end of the session, participants were asked to stop the car and leave the simulator. The experimenter removed electrodes and the participants could fill in the last part of the questionnaire. Then, they were thanked and discharged.

#### 4.1.5. Pilot Study

Eight people took part in the pilot study. The purpose was to check that all the data were correctly recorded (physiological signals and driving data) and that the driving scenario was running flawlessly. Shadows on the lane due to the reflection of sunlight on trees were removed from the driving environment. Indeed, obstacles were not triggered at the same location for all participants to minimize the impact of the visibility of drivers during the takeover situations. Also, the original design contained a third experimental condition. This condition was to count backwards by step of 13 to induce a higher cognitive load. However, we realized that it was too demanding to perform this task for 20 min.

#### 4.1.6. Statistical Analysis

The analyses were performed using IBM SPSS Statistics 25. By examining the audio files recorded during the execution of the NDRT, we found that the calculation task was performed correctly and accurately. Four participants made some errors during the NDRT. Three of them made a mistake in the transition from 3,001 to 2,999, starting again from 3,999. The fourth participant obtained an even score at the end. However, they were not removed from the analysis because they kept counting, which was the most important for the inducement of MWL. For the subjective ratings of the NASA-TLX, nine participants were removed due to due to issues with the online questionnaire. To test for the difference of MWL between the control group and the treatment group, analyses of variances (ANOVAs) were calculated for each questionnaire item and the global score of MWL. Cohen's effect size is reported when the ANOVA showed a significant result.

### 4.2. Classification Method

This section describes the methodology used to classify drivers' condition (secondary task vs. no secondary task) based on the recorded physiological signals. A first goal was to investigate the effect of sensor fusion on classification accuracy. The classification was performed for each signal independently (ECG, EDA, RESP), each possible pair of signals and all signals combined. A second goal was to observe the effect of segmentation level. In other words, the main driving session was segmented into windows of different size that were used to compute features. Six segmentation levels were tested : 1, 2, 5, 10, 20, and 40. With a segmentation level of 1, the features were computed from one 20-min window, whereas a segmentation level of 40 consisted of 40 30-s windows for computing features. The higher the segmentation level was, the more training examples the algorithm had for training. This process aimed at investigating the shortest time required to record physiological parameters to classify accurately the MWL of drivers. Overall, this work will help to find the best trade-off between the number and type of physiological signals needed, the optimal time-span for recording physiological data and the performance of a model to classify the level of MWL workload, with the ultimate goal of implementing such model in future automated vehicles under real-world conditions.

#### 4.2.1. Data Preprocessing

The preprocessing of raw physiological data was automated using the Neurokit library in Python (Makowski et al., 2021). Neurokit is a module that provides high-level integrative functions to process and exploit bio-signals. Signals from the baseline and driving phases were processed separately. The result of the processing step resulted in the computation of physiological indicators. To summarize the indicators computed in this study, a definition of each indicator calculated from physiological raw signals is proposed in Table 2.

**Table 2:** Summary of physiological indicators computed from raw physiological signals.

**Signal**	**Indicator**	**Domain**	**Description**
EDA	Mean raw level		The mean value of filtered EDA signal
	Min raw value		The minimum value of filtered EDA signal
	Max raw value		The maximum value of filtered EDA signal
	Std raw value		The standard deviation of filtered EDA signal
	Mean tonic level		The mean value of tonic EDA signal
	Max tonic value		The minimum value of tonic EDA signal
	Min tonic value		The maximum value of tonic EDA signal
	Std tonic value		The standard deviation of tonic EDA signal
	Amp. NS-SCRs		The mean amplitude of NS-SCRs (computed from phasic EDA signal)
	Freq. NS-SCRs		The number of NS-SCRs per minute (computed from phasic EDA signal)
ECG/RESP	Mean Rate	Time	The mean number of cardiac cycles per minute
	Mean		The mean time of IBIs/BBs
	Median		The median of the absolute values of the successive differences between adjacent IBIs/BBs
	MAD		The mean absolute deviation of IBIs/BBs
	SD		The standard deviation of IBIs/BBs
	SDSD		The standard deviation of the successive differences between adjacent IBIs/BBs
	CV		The Coefficient of Variation, i.e., the ratio of SD divided by Mean
	mCV		Median-based Coefficient of Variation, i.e., the ratio of MAD divided by Median
	RMSSD		The square root of the mean of the sum of successive differences between adjacent IBIs/BBs
	CVSD		The coefficient of variation of successive differences; the RMSSD divided by Mean
	LF	Frequency	The spectral power density pertaining to low frequency band (0.04 to 0.15 Hz)
	HF		The spectral power density pertaining to high frequency band (0.15 to 0.4 Hz)
	LF/HF		The ratio of low frequency power to high frequency power
	SD1	Non-linear	Measure of the spread of IBIs/BBs on the Poincaré plot perpendicular to the line of identity
	SD2		Measure of the spread of RR intervals on the Poincaré plot along the line of identity
	SD2/SD1		Ratio between long and short term fluctuations of IBIs (SD2 divided by SD1)
ECG	pNN50	Time	The proportion of successive IBIs greater than 50 ms, out of the total number of IBIs
	pNN20		The proportion of successive IBIs greater than 20 ms, out of the total number of IBIs
	TINN		The baseline width of IBIs distribution obtained by triangular interpolation
	HTI		The HRV triangular index (total number of IBIs divided by the height of IBIs histogram)
	VHF	Frequency	Variability, or signal power, in very high frequency (0.4–0.5 Hz)
	LFn		The normalized low frequency, obtained by dividing the low frequency power by the total power
	HFn		The normalized high frequency, obtained by dividing the low frequency power by the total power
	LnHF		The log transformed HF
	CSI	Non-linear	The Cardiac Sympathetic Index (longitudinal variability of Poincaré plot divided by transverse variability)
	CVI		The Cardiac Vagal Index (logarithm of the product of longitudinal and transverse variability)
	CSI_modified		The modified CSI (the square of the longitudinal variability divided by transverse variability)
RESP	Mean amplitude	Time	The mean respiratory amplitude
	ApEn	Non-linear	The approximate entropy of RRV
	DFA2		A long-term fluctuation value. Only be computed if mora than 640 breath cycles in the signal
RSA	Mean		Mean of RSA estimates
	Mean Log		The logarithm of the mean of RSA estimates
	SD		The standard deviation of all RSA estimates
	NoRSA		The number of breath cycles from which RSA could not be calculated
	RSA_PB		The Porges–Bohrer estimate of RSA, optimal when the signal to noise ratio is low, in ln(ms^2)

*Identical indicators computed from both ECG and respiration (RESP) signal are grouped together. IBIs refers to interbeat intervals (ECG) and BBs refers to breath-to-breath cycles (RESP)*.

The EDA signal was processed using methods of convex optimization (Greco et al., 2016), which defines EDA as the sum of three terms: the phasic component, the tonic component, and an additive white Gaussian noise term incorporating model prediction errors as well as measurement errors and artifacts. To be able to process the EDA signal with the convex optimization method, it had been down-sampled to 50 Hz to reduce computation time. The signal had also been filtered with a Finite Impulse Response low-pass filter of fourth order with a cut-off frequency of 5 Hz and smoothed using the convolution of a filter kernel with the input signal (Smith, 1999). That smoothing process used the moving average principle, with a window size of three-quarters the sampling rate. The output was the EDA raw signal, the filtered signal, the tonic component, the phasic component, the SCR onsets, peak indexes and amplitudes. Based on the related work, we chose to use the filtered signal, the tonic component and indicators that characterize NS-SCRs because we evaluate changes in drivers' state over a long period. Hence, EDA indicators including the minimum, maximum, standard deviation and mean values of filtered and tonic EDA signals were computed in this study, in addition to the frequency and the mean amplitude of NS-SCRs.

The ECG signal was filtered with a Finite Impulse Response band-pass filter of fourth-order with cut-off frequencies of 3 and 45 Hz. A QRS-detector algorithm was used to locate R-peaks from the ECG signal (Hamilton, 2002). The output was the ECG raw signal, the filtered signal and the R-peaks indexes. From that, HR and HRV indicators were computed. HRV indicators included time domain, frequency domain and non-linear domain indicators.

The respiration signal was filtered with a Butterworth band-pass filter of second-order with cut-off frequencies of 0.1 and 0.35 Hz and smoothed with the same process than EDA and a rectangular window size (also known as Dirichlet window) of 3 s (Smith, 1999). The output was the respiration raw signal, the filtered signal, the respiratory cycles onsets, and respiratory phases (inspirations and expirations). From that, indicators of rate and variability of respiration were computed.

Also, from both respiration and ECG signal, RSA features were computed using the peak-to-trough (P2T) and Porges–Bohrer methods. The P2T algorithm computes all RSA estimates in a given period. For each breath, an estimate of RSA is calculated by subtracting the shortest heart period during inspiration from the longest heart period during a breath cycle (Lewis et al., 2012). RSA features included the mean, the standard deviation and the logarithm of the P2T estimates (in milliseconds), in addition to a measure computed with the Porges–Bohrer method, as explained in Lewis et al. (2012).

#### 4.2.2. Feature Generation and Normalization

The same feature engineering process was applied to data of all participants, regardless of their experimental condition. Each indicator presented above was calculated for each segment of the driving phase. It was taken as a feature for the classification. For each indicator, an additional feature was computed, corresponding to the difference of that indicator between the driving segment and the baseline. That feature engineering process aimed at taking into account the physiological state of participants at rest and evaluate the individual changes on each indicator during the driving session (Darzi et al., 2018). In this way, the model performance should be higher for a between-participant validation procedure, since the ultimate goal is to build a model that would perform well with any subject inside the car. In total, the three raw physiological signals served to compute 122 features corresponding to 61 physiological indicators (10 from EDA, 27 from ECG, 19 from RESP, five from RSA). For classifiers sensitive to the range of features, data were normalized using the maximum and minimum of each feature during a driving segment. The minimum value was subtracted to the feature value and then divided by the difference between the maximum and minimum values.

#### 4.2.3. Feature Selection

Statistical analysis techniques are usually employed to check for the effect of a between-subjects factor on dependant variables. Therefore, we chose to do an ANOVA on each one of the 122 features independently. Only the physiological features that reached the significance level (*p*-value lower than 0.05) were used for classification. The number of features was not the same depending on the segmentation level and the physiological signals used for the classification task.

#### 4.2.4. Selected Algorithms

At this step of the procedure, the dataset consisted of some selected features that were used as input of classifiers for the training and validation procedure. Three algorithms were selected based on results from previous research in the field (Son et al., 2013; Solovey et al., 2014; Darzi et al., 2018) and for their ease of implementation. They have been implemented in Python using the scikit learn machine-learning framework (Pedregosa et al., 2011). The effect of selected physiological signals and segmentation level was tested with each classifier. Their classification principle is detailed below:

***Random Forest Classifier (RF):***A random forest is a meta estimator that fits some decision tree classifiers on various sub-samples of the dataset and use averaging to improve the predictive accuracy and control over-fitting (Breiman, 2001).

***C-Support Vector Classifier (SVC):***The support vector classifier uses boundaries (linear or more complex) to separate data in the input feature space. The separation boundary is defined by a kernel. In this experiment, we tested four different kernels: the linear, the sigmoid and the polynomial ones, as well as the radial basis function (Hsu et al., 2010).

***Multi-Layer Perceptron Classifier (MLP):***A multi-layer perceptron consists of a set of nodes distributed in a number of layers. It contains at least three layers of nodes: an input layer, a hidden layer and an output layer. Except for the input nodes, each node is a neuron that uses a non-linear activation function. The multi-layer perceptron utilizes backpropagation as a supervised learning technique for training (Hastie et al., 2009). Here, we use the multi-layer perceptron as a classifier, meaning that the output layer contains only two nodes that output the probability that the driver was performing a secondary task or not, based on input features. The classifier contained one hidden layer and we only tested to change the number of neurons in that hidden layer.

#### 4.2.5. Optimization and Validation

To maximize the performance of classifiers, an optimization of hyperparameters of the three selected classifiers was done. The hyperparameter search aims to find the set of hyperparameters that minimizes the loss and maximizes the classification accuracy (Claesen and De Moor, 2015). The grid search technique was chosen to search for the best set of hyperparameters. It consists of predefining a range of values to test for each hyperparameter. The classifier tests all possible combinations of parameters for training and validation procedures. A first iteration of that grid search technique (GridSearch_1_) was performed with a wide range of values. The goal was to eliminate values of hyperparameters for which the model does not perform well and hence reduce this range for the final optimization during the validation procedure. It was done on the entire dataset which was split into a training set (75% of samples) and a validation set (25% of samples). The hyperparameters that have been tested during this first optimization procedure can be found in Table 3. The definition and the chosen range of values for each parameter are presented. This first procedure was done for each level of segmentation and the feature selection process was also applied. The second hyperparameter optimization process was done during the final validation procedure. It used a reduced range of values defined after the first optimization. The k-fold cross-validation method was select to validate the performance of classifiers and prevent classifiers from overfitting the data (Hastie et al., 2009). In this procedure, the dataset was split into 10-folds. Classifiers were trained using data from 9 subsets and then validated on the remaining subset. The validation was repeated 10 times, with each subset acting as the validation subset once. The second step of optimization with a refined range of parameters (GridSearch_2_) was performed within the final validation pipeline. The 10-fold validation procedure was performed once for each set of hyperparameters. Graphs and tables report results for the set of hyperparameters that gave the best mean accuracy for the classification overall 10 subsets.

**Table 3:** Tweaked hyperparameters during the first iteration of the grid search procedure (GridSearch_1_), with chosen ranges and step values for each parameter.

**Classifier**	**Parameter name**	**Parameter definition**	**Range**
RF	n_estimators	Number of trees in the forest.	[10, 507, 1,005, 2,000]
	max_features	Number of features to consider when looking for the best split.	Sqrt
	max_depth	Maximum depth of the tree. If None, then nodes are expanded until all leaves are pure or until all leaves contain less than 2 samples.	[None, 10, 57, 105, 152, 200]
SVC	kernel	Specifies the kernel type to be used in the algorithm	[linear, RBF]
	C	Regularization parameter.	[2e-3, 2e-1, 2e1, 2e7, 2e9, 2e11]
	gamma	Kernel coefficient for RBF kernel.	[2e-13, 2e-9] by step of 10
MLP	solver	Solver used for weight optimization.	[lbfgs, adam]
	max_iterations	Maximum number of iterations. Solver iterates until convergence or number of iterations.	[500, 1,500]
	alpha	L2 penalty (regularization term) parameter.	[1e-4, 1] by step of 10
	hidden_layer_sizes	The number of neurons in the hidden layer.	[32, 64, 128, 256, 512]
	random state	Determines random number generation for weights and bias initialization.	[0, 42]

*RBF refers to Radial Basis Function*.

## 5. Results

### 5.1. Statistical Validation of MWL Inducement

#### 5.1.1. Engagement on Task

The indicator used to check for the engagement on task was the frequency of orally spoken numbers. The participants counted backward, on average, to the number 2,740 (*M* = 2740.03, SD = 311.28), making an average of 452 (*M* = 452.49, SD = 155.64) calculations throughout the driving session. It is equivalent to 22.6 numbers orally spoken per minute, e.g., approximately one number every 3 s. During the experiment, the experimenters asked participants to resume counting on average twice (*M* = 2.00, SD = 1.77).

#### 5.1.2. Subjective Ratings (NASA-TLX)

**Figure 4:**
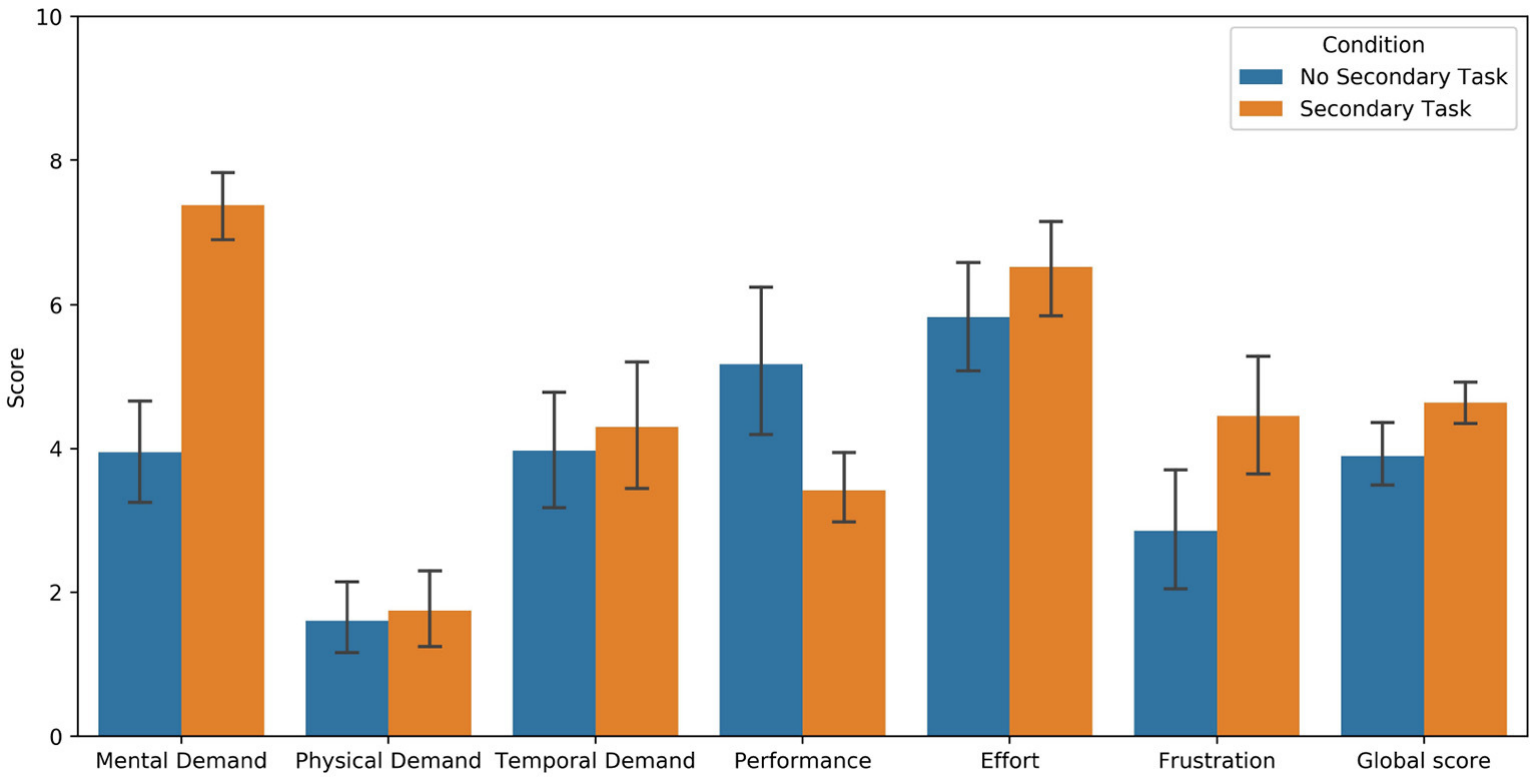
Subjective ratings of MWL collected from the NASA-TLX questionnaire. Low value means high performance for the *Performance* item.

To control the success of the MWL manipulation, subjective ratings of workload collected from the NASA-TLX questionnaire were used. Results indicate higher level of reported MWL for the group that performed the secondary task (*M* = 4.64, SD = 0.90) compared to the control group (*M* = 3.90, SD = 1.42; *F*_(1, 79)_ = 7.77, *p* < 0.05, *d* = 0.63), regarding the global score of the NASA-TLX. The difference was also significant between both groups for mental demand (*F*_(1, 79)_ = 59.85, *p* < 0.001, *d* = 1.73), performance (*F*_(1, 79)_ = 9.07, *p* < 0.05, *d* = 0.67) and frustration (*F*_(1, 79)_ = 6.83, *p* < 0.05, *d* = 0.58). Means and standard deviations for all components of the questionnaire are shown in Figure 4.

### 5.2. Classification of Drivers' Workload

#### 5.2.1. Reduction of Hyperparameter Range

The first iteration of the grid search (GridSearch_1_) gave insights about the influence of hyperparameter values on the performance of the model. The RF classifier obtained the poorest results with 2,000 estimators and a maximum depth of 200. For the final pipeline, we reduced the range for these two parameters. The SVC classifier performed best across all segmentation levels with the linear kernel and *C*-values of 2e-1, 2e1, and 2e7. Therefore, we chose to only use the linear kernel and refine the final range of C-values. For some segmentation levels, the MLP classifier did not converge to achieve the best score after 1,500 iterations. Therefore, it was set to 2,000 for the final pipeline. The lbfgs solver (which stands for Limited-memory Broyden–Fletcher–Goldfarb–Shanno) was selected for the final optimization process because it gave better results more often than the adam solver. The smallest alpha value (1e-4) did not show satisfying results so it was excluded from the final range of values. The number of neurons in the hidden layer did not have much influence (except for 512 neurons). Therefore, a similar range of values was chosen. Finally, the random state was set at 42 for challenging the model with random initialization of weights and biases during the final procedure. The chosen range and step values for each hyperparameter tested during the final optimization procedure (GridSearch_2_) are summarized in Table 4.

**Table 4:** Final range of values tested for each hyperparameter (GridSearch_2_).

**Classifier**	**Parameter name**	**Range**
RF	n_estimators	[10, 257, 505, 752, 1,000]
	max_features	sqrt
	max_depth	[None, 10, 40, 70, 100]
SVC	kernel	linear
	C	[2e-3, 2e7] by step of 10
MLP	solver	lbfgs
	max_iterations	2,000
	alpha	[1e-3, 1] by step of 10
	hidden_layer_sizes	[32, 64, 128, 256]
	random state	42

#### 5.2.2. Influence of the Number of Selected Physiological Signals

**Figure 5:**
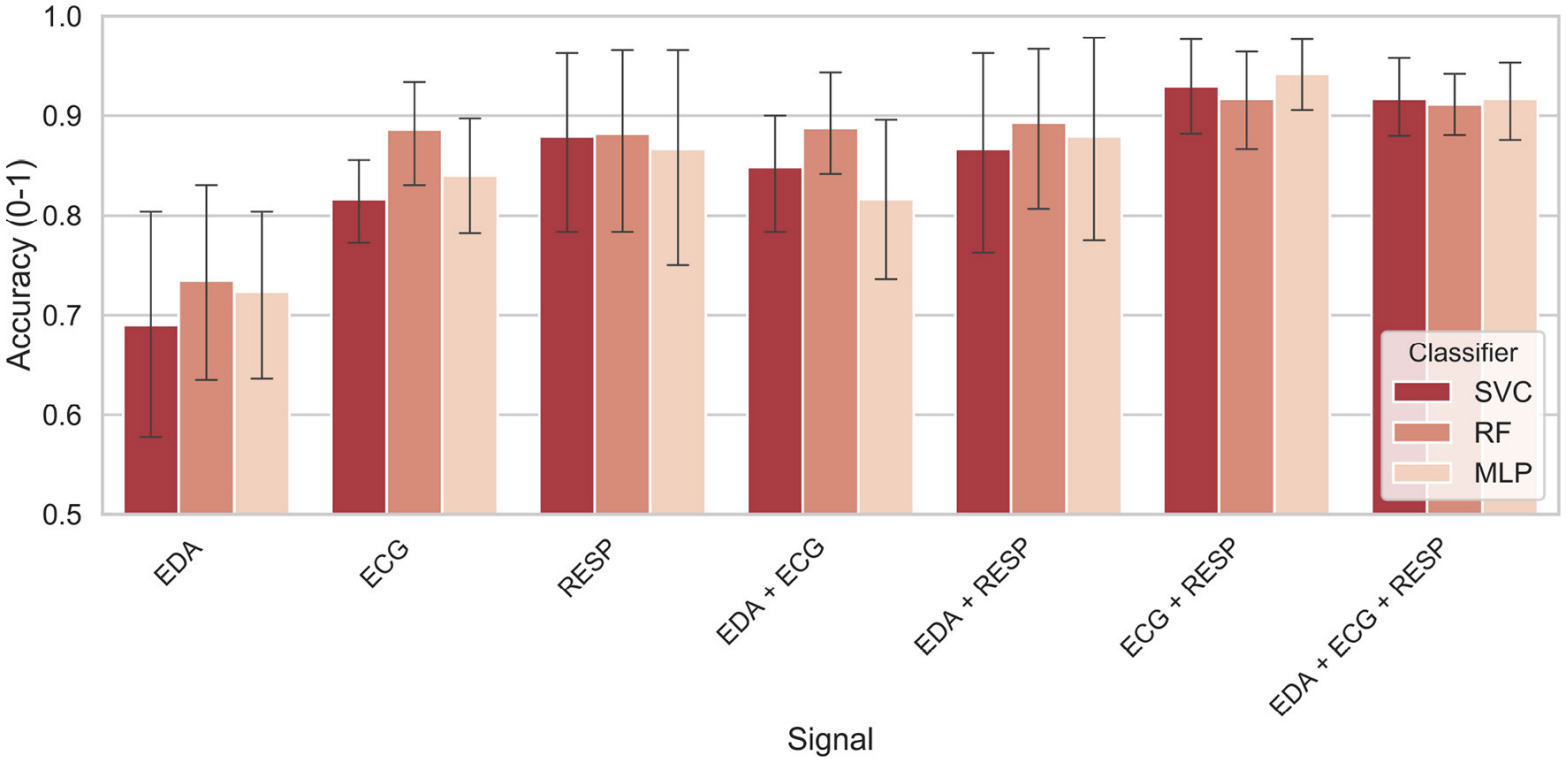
Classification accuracy as a function of selected physiological signals and classifier.

Figure 5 shows the means and standard deviations of the classification accuracy using the 10-fold validation procedure. Results are reported for each classifier depending on the type and the number of chosen physiological signals, with a segmentation level of 1. For each combination of selected signals, Table 5 shows the best mean accuracy (and standard deviation) and the classifier which performed best to classify drivers' condition over the 10-folds. Using only EDA as the input signal, the model showed the lowest performance, achieving between 69 and 73% accuracy regardless of the classifier. The model with ECG alone achieved 82–89% accuracy. With only one physiological signal as the input of classifiers, the respiration achieved the best results with an accuracy close to 90% on average over the 10-folds. With two signals as the input of classifiers, the combination of EDA and ECG features showed the lowest accuracy, between 82 and 89% accuracy. The combination of EDA and respiration as input signals gave 87–89% accuracy. The best combination of two signals was respiration and ECG, achieving 92–94% accuracy depending on the selected classifier. Finally, the combination of the three signals resulted in accuracy between 91 and 92% for classifying drivers' condition.

**Table 5:** Best score for each combination of selected signals (with a segmentation level of 1).

**Selected signal**	**Best classifier**	**Best accuracy [Mean (SD)]**
EDA	RF	0.73 (0.15)
ECG	RF	0.89 (0.09)
RESP	RF	0.88 (0.15)
EDA + ECG	RF	0.89 (0.09)
EDA + RESP	RF	0.89 (0.13)
**ECG + RESP**	**MLP**	**0.94 (0.06)**
EDA + ECG + RESP	SVC/MLP	0.92 (0.09)

*Bold values show the best score achieved by the model*.

#### 5.2.3. Influence of the Segmentation Level

**Figure 6:**
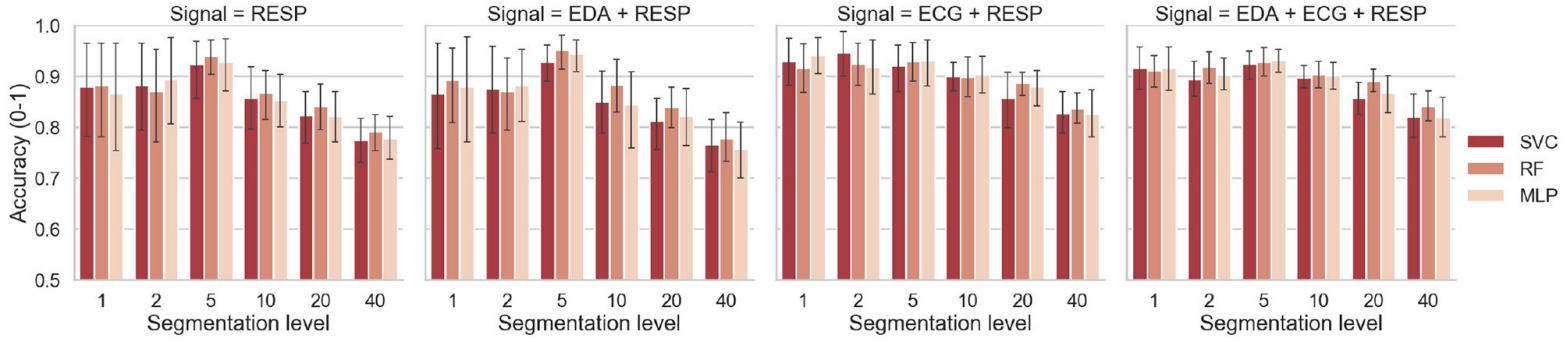
Classification accuracy as a function of the segmentation level and classifier.

For each classifier and each segmentation level from 1 to 40, Figure 6 shows the means and standard deviations achieved by the model after the classification task. The results are reported only for selected signals that achieved accuracy over 85% with a segmentation level of 1 with at least two classifiers. It includes the respiration alone, both pairs of EDA with respiration and ECG with respiration, and the fusion of the three signals. Best results for each level of segmentation are summarized in Table 6. The classifier and the combination of signals that gave the best results are also reported.

**Table 6:** Best score for each segmentation level, with corresponding signals and classifier.

**Segmentation level**	**Best selection of signals**	**Best classifier**	**Best accuracy [Mean (SD)]**
1	ECG + RESP	MLP	0.94 (0.06)
2	ECG + RESP	SVC	0.95 (0.07)
**5**	**EDA + RESP**	**RF**	**0.95 (0.05)**
10	ECG+RESP/EDA+ECG+RESP	SVC	0.90 (0.05)
20	ECG+RESP/EDA+ECG+RESP	RF	0.89 (0.04)
40	ECG+RESP/EDA+ECG+RESP	RF	0.84 (0.05)

*Bold values show the best score achieved by the model*.

## 6. Discussion

### 6.1. Manipulation of Workload

Regarding the results from the experimental manipulation, measures of task performance showed that participants were sufficiently involved in the NDRT they were asked to perform. Indeed, they counted orally with a rate of one number every 3 s on average. Subjective ratings of MWL showed that participants in the NDRT condition reported a significantly higher level of MWL than participants in the control group. Mental demand was the component that showed the largest effect size. Results from task performance and subjective ratings indicate that the manipulation of MWL of participants was successful. We can hence consider that performing such speech-based NDRT for 20 min in conditionally automated driving is increasing the MWL of drivers. From that, the effect of a higher level of MWL on the collected physiological data of drivers can be analyzed. A procedure using machine learning techniques for classifying drivers' MWL was used and an interpretation of results is proposed below.

### 6.2. Interpretation of Results Depending on the Selected Signals

The results are first interpreted for the effect of selected physiological signals on classification performance. Features were computed with a segmentation level of 1, meaning that physiological indicators were calculated from the entire driving period (20 min). With only one physiological signal selected as an input of the model, results showed that the model was performing poorest when the only EDA signal was selected. Using ECG alone, the model performed best, achieving an accuracy of 89% with the RF classifier. However, from the three physiological signals alone, the model showed more consistent results across classifiers with the respiration signal selected alone as input. Indeed, each of the three classifiers achieved 87–88% of accuracy, but with higher variance compared to ECG. We can consider that both features computed independently from the respiration and ECG signals are useful to distinguish the driver conditions (verbal secondary task vs. no secondary task).

If we now look at the effect of sensor fusion on classification results, the fusion of EDA and ECG did not give better results than the ones achieved with ECG alone. In the same way, the fusion of EDA and respiration signals was not better than respiration alone. In previous studies, EDA indicators such as mean skin conductance level were shown to be sensitive to an increase of MWL (Engström et al., 2005; Mehler et al., 2009). Similar indicators were computed in this work such as mean tonic and raw level of EDA. Additional indicators relating the long-term changes of driver's state such as the frequency of NS-SCRs were supposed to improve the accuracy of the system. Results suggest that EDA features were the least useful ones to classify drivers' condition, as found by Haapalainen et al. (2010) and Son et al. (2013). Nevertheless, the model can achieve 73% with EDA features, which confirms that drivers' skin conductance is affected by the performance of a secondary task involving a verbal function (Engström et al., 2005; Collet et al., 2009; Mehler et al., 2009).

However, the model benefited from the fusion of two sensors without EDA. Indeed, the fusion of respiration and ECG signals showed to increase the accuracy of the system compared to the respiration or ECG alone, achieving accuracy levels of over 90% using all classifiers. This is consistent with statements made above, confirming that the ECG and respiration features are useful for classification. Also, the variance of scores obtained over the 10-folds was lower. This suggests that the performance of classifiers varied less from one-fold to the other during the classification task, making the model more robust. Besides, the fusion of the three signals as inputs of the model performed similarly (or slightly worse) than the one of respiration and ECG. The variance and accuracy achieved were also similar regardless of the classifier. Overall, the fusion of ECG and respiration showed to achieve the best performance to specify the drivers' condition, with an accuracy of 94% and a standard deviation of 0.06 across the 10-folds with the MLP classifier (Table 5). It is probably due to the additional respiratory sinus arrhythmia features that were computed during the processing of ECG and respiration signals. These features were taken into account in the classification procedure and might have helped the model to capture more information about the change of phase between ECG and respiration signals during the execution of the task.

If we compare the results to reviewed studies reported in Table 1, the accuracy achieved in this study is better, using only physiological features for the classification. Still, results must be compared carefully since the experimental settings varied from one study to another: the driving environment, the task to complete or the classification procedure. The tasks performed by participants in previous studies were either visual or auditory. The only study in which the task was similar to the task administered in our experiment is the one led by Solovey et al. (2014). Participants had to perform the auditory n-back task and answer verbally to targets. Overall, if we compare our results with those of the latter study based on accuracy measurement, a better accuracy was achieved in this work, probably because the features were calculated over a 20-min time window.

### 6.3. Interpretation of Results for the Effect of Segmentation Level

In this study, the effect of segmentation on the performance of the model was also investigated. For each driver, the physiological signals collected during the driving session were split into several parts (from 1 to 40) and physiological indicators were computed for each segment. Regardless of the classifier and the chosen physiological signals, increasing the segmentation level from 1 to 5 showed to increase the accuracy of the model. Especially for respiration alone and respiration with EDA signals, the model gained around 10% of accuracy, as shown on Figure 6. For these signals that gave a lower accuracy with a segmentation level of 1, the model benefited from sensor fusion. The features computed on 4-min time windows (segmentation of 5) were more accurate to depict the condition of drivers. For segmentation levels of 5–40, increasing the segmentation level showed to decrease the accuracy, regardless of the selected signals. Even if the model had more training example for the classification task, it was more difficult to predict the driver's condition when the features were computed on time windows shorter than 4 min. However, even with 30-s time windows, the model was still able to achieve 84% accuracy with both ECG and respiration and the three signals (Table 6). For small time windows, Solovey et al. (2014) also found that enlarging the time window used for computing features (e.g., decreasing the segmentation level) increases the accuracy of the model. Again, if we compare our results with those of the latter study based on accuracy measurement, a lower accuracy was obtained in our study over 30-s time windows (84 vs. 90%). However, they used sliding windows to compute features so their model probably had more training data than our model to maximize its performance. Finally, another interesting result is that increasing the segmentation level showed to reduce the variance for some pairs of signals (error bars on Figure 6). This would suggest that the model could be more robust if a reduced time window is used for evaluating driver's state.

### 6.4. Selection of Best Trade-Off Between Performance and Number of Physiological Signals

For an implementation of a model able to classify drivers' MWL in future automated cars, the goal is to select the best trade-off between the length of the time window used to compute features from the physiological signals and the performance of the system. Based on results obtained in this study, we would select a time window of 4 min to compute features since that segmentation level gave the best accuracy with low variance. For the selection of signals, three options would be possible based on results obtained in this work. The first option would be to choose only the respiration alone as input signal. It would facilitate the implementation of such model under real-world conditions since only one sensor would be necessary to detect driver's MWL with a high accuracy (over 90%). The respiration could be measured either using non-contact respiratory monitoring methods (Min et al., 2010; Al-Khalidi et al., 2011) or contact-based methods using a piezoelectric sensor mounted in the seat belt. The second option would be to select the three signals, because it showed the lowest variance over the 10-folds, meaning that the prediction in real-time of driver's condition would be more reliable from one time to the next. If EDA and ECG would be selected as inputs signals in addition of RESP, both signals could be collected from an intelligent watch or from sensors integrated in smart garments (Sonderegger, 2013; Schneegass et al., 2015). The third option would be to use EDA and respiration signals, that showed the highest performance (high accuracy and low variance) in the present study. In practice, recent advances in technology allow for a continuous recording of the EDA and ECG signals. EDA can be collected from wearable devices such as watches, but they might not give measures as sensitive as the ones obtained with the gold-standard sensors used in this experiment. This can be explained by the lower sensitivity of the wrist tissue and the lower density of eccrine glands in this area compared to the volar surface of the hands (palms) or the foot (sole) (Taylor and Machado-Moreira, 2013). Besides, watches are currently using a plethysmograph sensor and do not provide fine-grained HRV features. However, we must consider that advances in wearable devices and smart clothes might give the possibility to collect robustly and continuously ECG, EDA and respiration signals in a near future. Technologies such as built-in sensors in the seat, radars or intelligent clothes with electrodes such as socks or chest strap are conceivable. Since, we cannot predict the pace of development of new technologies in the field of smart sensors and garments, we make here a proposition based on the empirical results of this work, taking into account the three physiological signals. Therefore, based on results obtained in this experimental study it can be argued that the combination of EDA and respiration signals with a time window of 4 min should be selected for an optimal prediction of orally induced MWL in conditionally automated driving. In previous studies, physiological indicators showed a great potential to detect an increase of driver's MWL due to the performance of a secondary task while driving manually. This study showed that it is also possible to use such indicators for distinguishing two different levels of driver's MWL at a higher level of automation. Previous findings on MWL evaluation can hence be considered in the specific context of automated driving (Level 3 or more according to the SAE taxonomy; SAE, 2018). Therefore, physiological sensors could be worn by drivers so that the car could evaluate their state continuously in conditionally automated driving. This evaluation of driver's state could be used by the car along with the evaluation of the driving situation to provide an optimal support to the driver through in-car interfaces. However, the successful implementation and acceptance of such algorithm depends on people's willingness to wear such sensors in the car. However, although highly interesting and challenging for the future development of the car industry, this is a different topic and not the subject of this paper.

### 6.5. Limitations and Further Research

There are several limitations that need to be discussed. A first limitation is that the verbal task might have influenced the respiratory pattern of subjects and therefore influenced our physiological indicators (Cacioppo et al., 2007). Therefore, based on the present findings, we can only state that a higher level of MWL induced by a continuous verbal task can be accurately detected in the context of conditionally automated driving. Future research needs to be conducted to investigate to what extent similar results could be obtained in situations of high MWL induced by a task that does not require the participants to speak. In addition, only a subset of all available features were used for the classification task. However, some features that were excluded could have been useful for the classification because of their correlation with other features. Therefore, different strategies for feature selection should be explored. Also, similar experiments should be conducted to collect physiological data from drivers performing cognitive tasks that involve other modalities. The visual and/or auditory n-back task (without verbal answer) could be used to manipulate the MWL of drivers, as done in previous studies (Mehler et al., 2009; Son et al., 2013; Hogervorst et al., 2014; Solovey et al., 2014). Therefore, the model developed as part of this study needs to be evaluated using other NDRTs.

Another stake for the emergence of driver's state systems under real conditions is to be able to evaluate MWL in real-time. Since, Solovey et al. (2014) obtained an accuracy of around 90% using sliding windows of 30 s, it would be interesting to test the effect of sliding windows on our data to generate more training examples and see if it increases the performance of the model. Further studies should focus on evaluating MWL on shorter epochs of cognitive task. The duration of the task performed by drivers in this study was rather long (20 min). Even if the segmentation of data was tested, it might have facilitated the model to achieve good results. Another experiment should be led with participants performing cognitive NDRTs on shorter periods. In this way, it would be closer to reality because drivers might not perform verbal task during 20 min. Based on the new collected data, the same model will be tested to see if it still performs well to predict the MWL level of drivers on shorter periods. If the model's performance decreases too much, the model will need to be refined. To do that, we could consider using model architectures that are efficient with temporal data such as recurrent neural networks. The perceptron used in the MLP classifier would be replaced by gated recurrent units or long-short term memory cells (Hochreiter and Schmidhuber, 1997).

## 7. Conclusion

The main contribution was to use machine learning techniques to specify drivers' condition (verbal task or no task). Three different classifiers along with sensor fusion and six levels of data segmentation were compared. Results show that the model was able to successfully classify the state of the driver with an accuracy of 95% using physiological features from two signals, computed from 4-min windows. The model benefited from sensors' fusion when the respiration and ECG were both selected as input signals. We also showed that increasing the segmentation level from 1 to 5 increased the performance of the classifiers, but increasing the segmentation level from 5 to 40 decreased the performance. For the concrete implementation of such a model under real driving conditions, a fusion of EDA and respiration signals with a time window of 4 min should be considered to compute physiological features in order to classify drivers' MWL in conditionally automated driving.

## Data Availability Statement

The datasets presented in this article are not readily available because the dataset is saved in a repository and used only in the context of the AdVitam project. Requests to access the datasets should be directed to Quentin Meteier, quentin.meteier@hes-so.ch.

## Ethics Statement

This study involving human participants was reviewed and approved by Ethics Committee of the Department of Psychology of the University of Fribourg. The participants provided their written informed consent to participate in this study.

## Author Contributions

EM, AS, LA, MW and OA developed the research question. QM and AS were responsible for study design and data collection. QM, SR and MC developed machine learning algorithms and conducted data analysis. QM and AS wrote the manuscript. QM and AS designed the experimental procedure and collected the data. QM and SR implemented the classification procedure. All authors contributed to the writing and revision processes.

## Conflict of Interest

The authors declare that the research was conducted in the absence of any commercial or financial relationships that could be construed as a potential conflict of interest.

## References

[B1] Aasman J. Mulder G. Mulder L. J. M. (1987). Operator effort and the measurement of heart-rate variability. Hum. Factors 29, 161–170. 10.1177/001872088702900204 3610181

[B2] Al-Khalidi F. Q. Saatchi R. Burke D. Elphick H. Tan S. (2011). Respiration rate monitoring methods: a review. Pediatr. Pulmonol. 46, 523–529. 10.1002/ppul.21416 21560260

[B3] Angelini L. Khaled O. A. Caon M. Mugellini E. Lalanne D. (2014). Hugginess: encouraging interpersonal touch through smart clothes, in ISWC '14 Adjunct (Seattle, WA). 10.1145/2641248.2641356

[B4] Baek H. Cho C.-H. Cho J. Woo J. (2015). Reliability of ultra-short-term analysis as a surrogate of standard 5-min analysis of heart rate variability. Telemed. J. e-Health 5, 404–414. 10.1089/tmj.2014.0104 25807067

[B5] Bernston G. G. Cacioppo J. T. Quigley K. S. (1993). Respiratory sinus arrhythmia: autonomic origins, physiological mechanisms, and psychophysiological implications. Psychophysiology 30, 183–196. 10.1111/j.1469-8986.1993.tb01731.x 8434081

[B6] Boucsein W. (2012). Electrodermal Activity. Springer US. 10.1007/978-1-4614-1126-0

[B7] Breiman L. (2001). Random forests. Mach. Learn. 45, 5–32. 10.1023/A:1010933404324

[B8] Brookhuis K. Waard D. Samyn N. (2004). Effects of mdma (ecstasy), and multiple drugs use on (simulated) driving performance and traffic safety. Psychopharmacology 173, 440–445. 10.1007/s00213-003-1714-5 14714102

[B9] Brookhuis K. A. De Waard D. (2001). Assessment of drivers' workload: performance, subjective and physiological indices, in Stress, Workload and Fatigue, P. A. Hancock and P. A. Desmond (Lawrence Erlbaum Associates), 321–333. 10.1201/b12791-2.5

[B10] Brookhuis K. A. de Waard D. (2010). Monitoring drivers' mental workload in driving simulators using physiological measures. Accident Anal. Prevent. 42, 898–903. 10.1016/j.aap.2009.06.001 20380918

[B11] Bulmer M. De Vaus D. A. Fielding N. (2004). Questionnaires. London; Thousand Oaks, CA: Sage Publications.

[B12] Cacioppo J. T. Tassinary L. G. Berntson G. G. (Eds.) (2007). Handbook of Psychophysiology, 3rd Edn. Cambridge University Press. 10.1017/CBO9780511546396

[B13] Camm A. J. Malik M. Bigger J. T. Breithardt G. Cerutti S. Cohen R. J.. (1996). Heart rate variability: standards of measurement, physiological interpretation and clinical use. Task force of the European society of cardiology and the north American society of pacing and electrophysiology. Circulation 93, 1043–1065. 8598068

[B14] Claesen M. De Moor B. (2015). Hyperparameter search in machine learning. arXiv preprint arXiv:1502.02127.

[B15] Collet C. Clarion A. Morel M. Chapon A. Petit C. (2009). Physiological and behavioural changes associated to the management of secondary tasks while driving. Appl. Ergon. 40, 1041–1046. 10.1016/j.apergo.2009.01.007 19249012

[B16] Darzi A. Gaweesh S. M. Ahmed M. M. Novak D. (2018). Identifying the causes of drivers' hazardous states using driver characteristics, vehicle kinematics, and physiological measurements. Front. Neurosci. 12:568. 10.3389/fnins.2018.00568 30154696 PMC6102354

[B17] De Waard D. (1997). The measurement of drivers' mental workload (Ph.D. thesis). Traffic Research Centre, University of Groningen, Haren, Netherlands.

[B18] Engström J. Johansson E. Östlund J. (2005). Effects of visual and cognitive load in real and simulated motorway driving. Transport. Res. F Traffic Psychol. Behav. 8, 97–120. 10.1016/j.trf.2005.04.012

[B19] Ferreira E. Ferreira D. Kim S. Siirtola P. Roning J. Forlizzi J. F.. (2014). Assessing real-time cognitive load based on psycho-physiological measures for younger and older adults, in 2014 IEEE Symposium on Computational Intelligence, Cognitive Algorithms, Mind, and Brain (CCMB) (Orlando, FL: IEEE), 39–48. 10.1109/CCMB.2014.7020692

[B20] Fisk A. D. Derrick W. L. Schneider W. (1986). A methodological assessment and evaluation of dual-task paradigms. Curr. Psychol. Res. Rev. 5, 315–327. 10.1007/BF02686599

[B21] Gawron V. J. (2019). Human Performance, Workload, and Situational Awareness Measures Handbook. CRC Press. 10.1201/9780429019562

[B22] Greco A. Valenza G. Lanata A. Scilingo E. P. Citi L. (2016). cvxEDA: a convex optimization approach to electrodermal activity processing. IEEE Trans. Biomed. Eng. 63, 797–804. 10.1109/TBME.2015.2474131 26336110

[B23] Haapalainen E. Kim S. Forlizzi J. F. Dey A. K. (2010). Psycho-physiological measures for assessing cognitive load, in Proceedings of the 12th ACM International Conference on Ubiquitous Computing - Ubicomp '10 (Copenhagen: ACM Press), 301. 10.1145/1864349.1864395

[B24] Hamilton P. (2002). Open source ECG analysis, in Computers in Cardiology (Memphis, TN: IEEE), 101–104.

[B25] Hart S. G. Staveland L. E. (1988). Development of NASA-TLX (task load index): results of empirical and theoretical research, in Advances in Psychology, Volume 52 of Human Mental Workload, eds P. A. Hancock and N. Meshkati (Amsterdam: North Holland), 139–183. 10.1016/S0166-4115(08)62386-9

[B26] Hastie T. Tibshirani R. Friedman J. (2009). The Elements of Statistical Learning: Data Mining, Inference, and Prediction, 2nd Edn. New York, NY: Springer-Verlag.

[B27] Hochreiter S. Schmidhuber J. (1997). Long short-term memory. Neural Comput. 9, 1735–1780. 10.1162/neco.1997.9.8.1735 9377276

[B28] Hogervorst M. A. Brouwer A.-M. van Erp J. B. F. (2014). Combining and comparing EEG, peripheral physiology and eye-related measures for the assessment of mental workload. Front. Neurosci. 8:322. 10.3389/fnins.2014.00322 25352774 PMC4196537

[B29] Hsu C.-W. Chang C.-C. Lin C.-J. (2010). A practical guide to support vector classification. Technical report.

[B30] Huigen E. Peper A. Grimbergen C. A. (2002). Investigation into the origin of the noise of surface electrodes. Med. Biol. Eng. Comput. 40, 332–338. 10.1007/BF02344216 12195981

[B31] Ikehara C. Crosby M. (2005). Assessing cognitive load with physiological sensors, in Proceedings of the 38th Annual Hawaii International Conference on System Sciences (Big Island, HI), 295a.

[B32] Kim H. Kim W. Kim J. Lee S.-J. Yoon D. (2019). A study on the effects of providing situation awareness information for the control authority transition of automated vehicle, in 2019 International Conference on Information and Communication Technology Convergence (ICTC) (Jeju Island), 1394–1396. 10.1109/ICTC46691.2019.8939867

[B33] Krueger E. Schneider A. Sawyer B. D. Chavaillaz A. Sonderegger A. Groner R.. (2019). Microsaccades distinguish looking from seeing. J. Eye Movement Res. 12, 1–14. 10.16910/jemr.12.6.2 PMC7962679 33828752

[B34] Le A. S. Aoki H. Murase F. Ishida K. (2018). A novel method for classifying driver mental workload under naturalistic conditions with information from near-infrared spectroscopy. Front. Hum. Neurosci. 12:431. 10.3389/fnhum.2018.00431 30416438 PMC6213715

[B35] Lewis G. F. Furman S. A. McCool M. F. Porges S. W. (2012). Statistical strategies to quantify respiratory sinus arrhythmia: are commonly used metrics equivalent? Biol. Psychol. 89, 349–364. 10.1016/j.biopsycho.2011.11.009 22138367 PMC3269511

[B36] Li N. Busso C. (2013). Analysis of facial features of drivers under cognitive and visual distractions, in 2013 IEEE International Conference on Multimedia and Expo (ICME) (San Jose, CA: IEEE), 1–6. 10.1109/ICME.2013.6607575

[B37] Makowski D. Pham T. Lau Z. J. Brammer J. C. Lespinasse F. Pham H.. (2021). Neurokit2: a python toolbox for neurophysiological signal processing. Behav. Res. Methods. 10.3758/s13428-020-01516-y 33528817

[B38] Malik M. Terrace C. (1996). Heart rate variability. Standards of measurement, physiological interpretation, and clinical use. Eur. Heart J. 17, 354–381. 10.1093/oxfordjournals.eurheartj.a014868 8737210

[B39] Mehler B. Reimer B. Coughlin J. (2012). Sensitivity of physiological measures for detecting systematic variations in cognitive demand from a working memory task. Hum. Factors 54, 396–412. 10.1177/0018720812442086 22768642

[B40] Mehler B. Reimer B. Coughlin J. Dusek J. (2009). The impact of incremental increases in cognitive workload on physiological arousal and performance in young adult drivers. Transport. Res. Rec. 2138, 6–12. 10.3141/2138-02

[B41] Merat N. Seppelt B. Louw T. Engstrom J. Lee J. D. Johansson E.. (2019). The Out-of-the-Loop concept in automated driving: proposed definition, measures and implications. Cogn. Technol. Work 21, 87–98. 10.1007/s10111-018-0525-8

[B42] Min S. D. Kim J. K. Shin H. S. Yun Y. H. Lee C. K. Lee M. (2010). Noncontact respiration rate measurement system using an ultrasonic proximity sensor. IEEE Sensors J. 10, 1732–1739. 10.1109/JSEN.2010.2044239

[B43] Mulder L. J. M. (1992). Measurement and analysis methods of heart rate and respiration for use in applied environments. Biol. Psychol. 34, 205–236. 10.1016/0301-0511(92)90016-N 1467394

[B44] Nakajima Y. Tanaka K. (2017). Effects of active and passive secondary tasks in a take-over situation during automated driving, in 2017 IEEE International Conference on Systems, Man, and Cybernetics (SMC) (Banff, AB), 1161–1166. 10.1109/SMC.2017.8122769

[B45] NHTSA (2017). Distracted Driving in Fatal Crashes (Traffic Safety Facts Research Note. National Center for Statistics and Analysis.

[B46] Parasuraman R. Sheridan T. Wickens C. (2000). A model for types and levels of human interaction with automation. IEEE Trans. Syst. Man Cybernet. A Syst. Hum. 30, 286–297. 10.1109/3468.844354 11760769

[B47] Pedregosa F. Varoquaux G. Gramfort A. Michel V. Thirion B. Grisel O.. (2011). Scikit-learn: machine learning in Python. J. Mach. Learn. Res. 12, 2825–2830. 10.5555/1953048.2078195

[B48] Petermeijer S. Bazilinskyy P. Bengler K. de Winter J. (2017). Take-over again: investigating multimodal and directional TORs to get the driver back into the loop. Appl. Ergon. 62, 204–215. 10.1016/j.apergo.2017.02.023 28411731

[B49] Pettitt M. Burnett G. Stevens A. (2005). Defining driver distraction, in Intelligent Transportation Society of America - 12th World Congress on Intelligent Transport Systems (San Francisco, CA), 5.

[B50] SAE. (2018). Taxonomy and Definitions for Terms Related to Driving Automation Systems for On-Road Motor Vehicles.

[B51] Salahuddin L. Cho J. Jeong M. G. Kim D. (2007). Ultra short term analysis of heart rate variability for monitoring mental stress in mobile settings, in 2007 29th Annual International Conference of the IEEE Engineering in Medicine and Biology Society (Lyon), 4656–4659. 10.1109/IEMBS.2007.4353378 18003044

[B52] Schlegel R. E. (1993). Chapter 17/21, Driver mental workload. Automot. Ergon. 359–382.

[B53] Schneegass S. Hassib M. Zhou B. Cheng J. Seoane F. Amft O.. (2015). Simpleskin: towards multipurpose smart garments, in Adjunct Proceedings of the 2015 ACM International Joint Conference on Pervasive and Ubiquitous Computing and Proceedings of the 2015 ACM International Symposium on Wearable Computers, UbiComp/ISWC'15 Adjunct (New York, NY: Association for Computing Machinery), 241–244. 10.1145/2800835.2800935

[B54] Shaffer F. Ginsberg J. (2017). An overview of heart rate variability metrics and norms. Front. Public Health 5:258. 10.3389/fpubh.2017.00258 29034226 PMC5624990

[B55] Siegenthaler E. Costela F. M. McCamy M. B. Di Stasi L. L. Otero-Millan J. Sonderegger A.. (2014). Task difficulty in mental arithmetic affects microsaccadic rates and magnitudes. Eur. J. Neurosci. 39, 287–294. 10.1111/ejn.12395 24438491

[B56] Smith S. W. (1999). The Scientist and Engineer's Guide to Digital Signal Processing. California Technical Pub., San Diego, CA.

[B57] Solovey E. T. Zec M. Garcia Perez E. A. Reimer B. Mehler B. (2014). Classifying driver workload using physiological and driving performance data: two field studies, in Proceedings of the 32nd Annual ACM Conference on Human Factors in Computing Systems - CHI '14 (Toronto, ON: ACM Press), 4057–4066. 10.1145/2556288.2557068

[B58] Son J. Oh H. Park M. (2013). Identification of driver cognitive workload using support vector machines with driving performance, physiology and eye movement in a driving simulator. Int. J. Precis. Eng. Manufactur. 14, 1321–1327. 10.1007/s12541-013-0179-7

[B59] Sonderegger A. (2013). Smart garments-the issue of usability and aesthetics, in Proceedings of the 2013 ACM Conference on Pervasive and Ubiquitous Computing Adjunct Publication, UbiComp '13 Adjunct (New York, NY: Association for Computing Machinery), 385–392. 10.1145/2494091.2495969

[B60] Taylor N. Machado-Moreira C. (2013). Regional variations in transepidermal water loss, eccrine sweat gland density, sweat secretion rates and electrolyte composition in resting and exercising humans. Extreme Physiol. Med. 2:4. 10.1186/2046-7648-2-4 23849497 PMC3710196

[B61] Wandtner B. Schomig N. Schmidt G. (2018). Effects of non-driving related task modalities on takeover performance in highly automated driving. Hum. Factors 60, 870–881. 10.1177/0018720818768199 29617161

[B62] Wickens C. D. (2008). Multiple resources and mental workload. Hum. Factors 50, 449–455. 10.1518/001872008X288394 18689052

[B63] Young M. S. Brookhuis K. A. Wickens C. D. Hancock P. A. (2015). State of science: mental workload in ergonomics. Ergonomics 58, 1–17. 10.1080/00140139.2014.956151 25442818

[B64] Zijlstra F. Doorn L. (1985). The Construction of a Scale to Measure Perceived Effort. Department of Philosophy and Social Sciences.270333

